# 1253. Antimicrobial Activity of Cefepime in Combination with Taniborbactam Against Clinical Isolates of Enterobacterales from 2018-2020 Global Surveillance

**DOI:** 10.1093/ofid/ofab466.1445

**Published:** 2021-12-04

**Authors:** Meredith Hackel, Mark G G Wise, Daniel F Sahm

**Affiliations:** 1 IHMA, Inc., Schaumburg, Illinois; 2 IHMA, Schaumburg, Illinois

## Abstract

**Background:**

Taniborbactam (formerly VNRX-5133) is a novel cyclic boronate-based broad-spectrum β-lactamase inhibitor with potent and selective direct inhibitory activity against both serine- and metallo-β-lactamases (Ambler Classes A, B, C and D). Taniborbactam restores the activity of cefepime against many difficult to treat organisms, including cephalosporin- and carbapenem-resistant Enterobacterales and *Pseudomonas aeruginosa*. The activity of the investigational combination cefepime-taniborbactam (FTB) and comparator agents was evaluated against clinical isolates of Enterobacterales from a 2018-2020 global surveillance study.

**Methods:**

MICs of cefepime with taniborbactam fixed at 4 µg/mL and comparators were determined following CLSI M07-A11 guidelines against 10,543 Enterobacterales. Isolates were from community and hospital infections collected from 259 sites in 56 countries in 2018-2020. Resistant phenotypes were based on 2021 CLSI breakpoints. A set of 827 isolates with meropenem MIC ≥4 µg/mL (n=421) or with cefepime and/or ceftazidime MIC ≥2 µg/mL (n=406) was evaluated for the presence of MBLs, KPC, ESBLs, and OXA-48 group genes via PCR and sequencing. Forty-eight isolates with FTB MIC values of 16 µg/mL or greater were interrogated by WGS.

**Results:**

Overall, 23.0% and 15.9% of isolates were nonsusceptible (NS) to cefepime and piperacillin-tazobactam (TZP), respectively (Table). FTB had potent activity against all Enterobacterales, with MIC_50/90_ values of 0.06/0.25 µg/mL and 99.5% inhibited at ≤8 µg/mL. FTB maintained activity against MBL-, KPC-, OXA-48 group, and ESBL-positive isolates (MIC_90_ range, 1 to >16 µg/mL; 80.5% to 100% inhibited at ≤8 µg/mL). Isolates with elevated FTB MICs had IMP-type enzymes, variation in the cefepime target (penicillin binding protein 3), permeability defects in combination with acquired β-lactamases, and/or possible up-regulated efflux.

Results Table

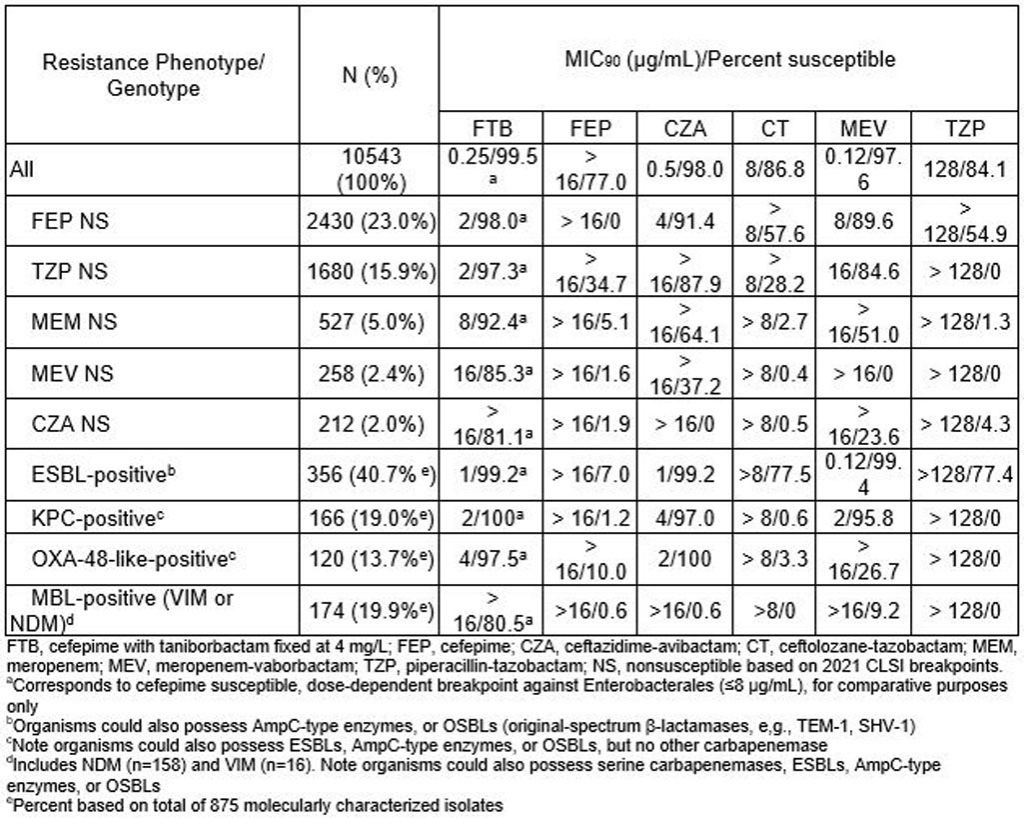

**Conclusion:**

Taniborbactam significantly restored the *in vitro* activity of cefepime against Enterobacterales, including isolates nonsusceptible to recently-approved BL/BLI combinations and expressing serine and metallo-β-lactamases. This support the continued development of FTB as a potential new treatment option for challenging infections due to resistant Gram-negative pathogens.

**Disclosures:**

**Meredith Hackel, PhD MPH**, **IHMA** (Employee)**Pfizer, Inc.** (Independent Contractor) **Mark G G. Wise, PhD**, **IHMA** (Employee)**Pfizer, Inc.** (Independent Contractor) **Daniel F. Sahm, PhD**, **IHMA** (Employee)**Pfizer, Inc.** (Independent Contractor)

